# Prediction and impact of maladaptive perfectionism on non-suicidal self-injury in adolescents: a study based on machine learning and structural equation modeling

**DOI:** 10.3389/fpsyt.2026.1714144

**Published:** 2026-03-05

**Authors:** Xin Wang, Tiejun Kang, Weiping Chen

**Affiliations:** 1 Second Hospital of Lanzhou University, Lanzhou, Gansu, China; 2 Tongji Hospital of Tongji University, Tongji University School of Medicine, Shanghai, China; 3 School of Psychology, Northwest Normal University, Lanzhou, Gansu, China

**Keywords:** adolescents, emotion regulation difficulties, impulsivity, maladaptive perfectionism, non-suicidal self-injury

## Abstract

**Background:**

Non-suicidal self-injury (NSSI) is highly prevalent among adolescents, yet the psychological mechanisms, particularly the synergistic effects of maladaptive perfectionism, impulsivity, and emotion regulation difficulties, remain inadequately understood. While recent longitudinal studies have focused on predicting NSSI occurrence using multiple machine learning algorithms, the current study addresses a complementary scientific question by examining the underlying psychological mechanisms through a cross-sectional multi-method approach.

**Methods:**

In a sample of 3,865 Chinese adolescents, we employed a multi-method approach combining machine learning (Support Vector Machine for classification) with structural equation modeling to analyze self-report data. It should be noted that due to the cross-sectional nature of our data, the relationships identified represent associations rather than causal effects.

**Results:**

The machine learning model demonstrated good discriminatory power for identifying NSSI (AUC = 0.79, 95% CI [0.76, 0.82]), with emotion regulation difficulties emerging as the strongest predictor. The chain mediation model revealed that maladaptive perfectionism is associated with NSSI through a sequential pathway: it is linked to heightened impulsivity, which in turn is associated with exacerbated emotion regulation difficulties, ultimately relating to self-injury.

**Conclusion:**

Maladaptive perfectionism is associated with risk for NSSI through a cascade of psychological processes involving impulsivity and emotion regulation deficits. These findings underscore the necessity for multi-target interventions that simultaneously address perfectionistic cognitions, impulsive tendencies, and regulatory skills in at-risk adolescents.

## 1 Introduction

Non-suicidal self-injury (NSSI) is a prevalent and clinically significant behavior among adolescents and young adults, defined as the intentional infliction of physical harm without suicidal intent. Its public health impact is substantial, with prevalence estimates around 23% in community youth and nearly 50% in hospitalized samples. NSSI is strongly associated with heightened risk for future self-injury, suicide attempts, and the development of severe psychopathology, including borderline personality disorder (1–4). Moreover, NSSI is increasingly recognized as a transdiagnostic risk marker for a wide spectrum of mental health difficulties, underscoring the urgent need for clearer mechanistic insights that can guide effective prevention and intervention efforts (5). Despite extensive documentation of its prevalence and consequences, the psychological processes that initiate and sustain NSSI remain insufficiently understood. In particular, there is a notable gap in integrative models that examine how maladaptive perfectionism, impulsivity, and emotion regulation difficulties interact to shape vulnerability to NSSI and contribute to its maintenance.

Maladaptive perfectionism involves rigid personal standards, harsh self-evaluation, and heightened concern about meeting perceived external expectations (6). Adolescence is a formative developmental period during which perfectionistic tendencies often intensify (7–9), with high levels observed across youth samples (10–12). Family dynamics and broader patterns of psychopathology further influence adolescents’ emotional and cognitive development (13–15). Rather than examining isolated associations between perfectionism and NSSI, the present study adopts an integrative perspective, conceptualizing maladaptive perfectionism as a trait vulnerability that interacts with impulsivity and emotion regulation deficits to elevate NSSI risk. Drawing on developmental and systems-oriented frameworks (16), we investigate how maladaptive perfectionism contributes to NSSI through intermediary psychological processes.

Impulsivity—defined as a tendency to act without adequate consideration of long-term consequences—has long been linked to self-injurious behaviors (17–19). Meta-analytic findings show that individuals who engage in NSSI frequently exhibit elevated impulsivity, especially in facets such as negative urgency and reduced perseverance (20, 21). Accordingly, we propose H1: impulsivity plays a mediating role in the association between maladaptive perfectionism and NSSI. This hypothesis is examined within a comprehensive analytic framework assessing direct, indirect, and chain-mediated pathways, positioning impulsivity as a potentially modifiable intervention target.

Emotion regulation difficulties represent another core component of NSSI vulnerability. Both the Experiential Avoidance Model (EAM) and the Emotional Cascade Model (ECM) posit that individuals may rely on maladaptive avoidance strategies to manage distressing emotions, which in turn reinforce self-injurious behavior (17). Adolescents who engage in NSSI often struggle with adaptive regulatory strategies such as cognitive refocusing and active coping (22). These persistent regulatory challenges—particularly when coupled with perfectionistic tendencies—suggest that deficits in emotion regulation may serve as a critical mechanism linking perfectionistic vulnerability to NSSI engagement.

**Figure 1:**
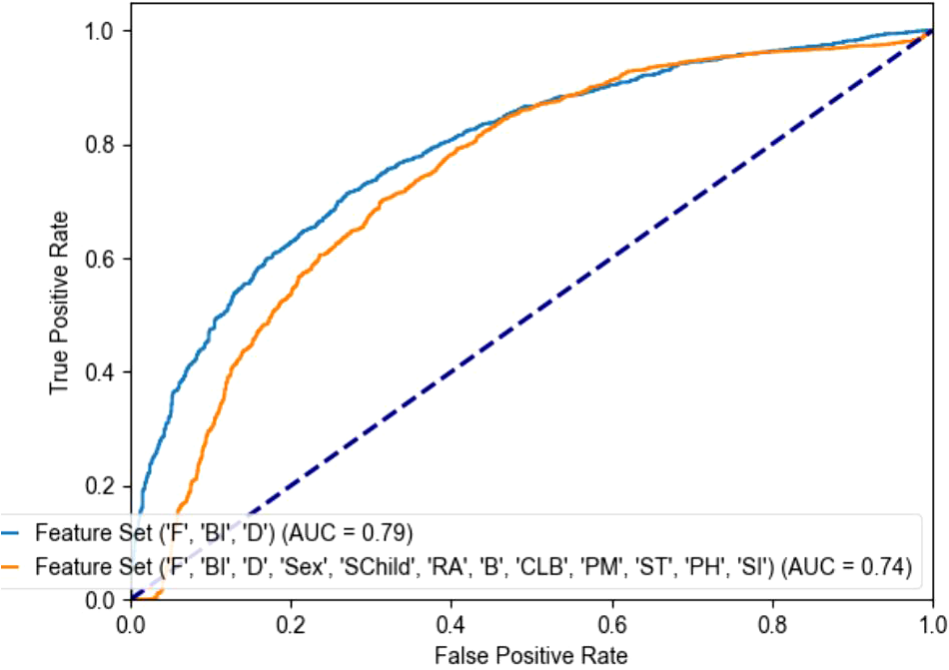
Receiver operating curve (ROC) of different feature combinations. F: CFMPS, measuring the cognitive, emotional, and behavioral characteristics typical of perfectionists, BI, BIS-11, measuring Impulsiveness; D, DERS, measuring Difficulties in Emotion Regulation; Schild, Single Child; RA, Residential Address; B, Board; CLB, Children Left Behind; PM, Parental Marriage; PH, Parental Health; SI, suicidal ideation.

The present study advances earlier machine learning work on NSSI prediction in two meaningful ways. First, although existing predictive models (e.g., Guo et al. (23)) have identified a range of broad risk factors, they have not directly addressed maladaptive perfectionism—a central dispositional vulnerability whose recognition as a key predictor meaningfully expands the nomological network of NSSI-related factors. Second, and perhaps more critically, our integration of machine learning with structural equation modeling moves the field beyond prediction alone by offering a clearer account of the mechanisms through which these risk factors operate. This dual “prediction–explanation” framework illustrates that maladaptive perfectionism contributes to NSSI not as an isolated trait, but through a sequential psychological process involving heightened impulsivity and subsequent emotion regulation difficulties. This study draws upon both the Emotional Cascade Model (24) and the Experiential Avoidance Model (17) to construct a chain mediation framework (**Figure 1**) that delineates how maladaptive perfectionism may contribute to NSSI. Specifically, we posit that individuals who hold rigid personal standards and experience an intense fear of failure are more likely to engage in persistent self-criticism and feel heightened pressure to meet those standards. This sustained internal tension may, in turn, manifest as impulsive tendencies (Path 1). Such impulsivity—particularly under conditions of negative affect (i.e., negative urgency)—can compromise the individual’s capacity to employ effective emotion regulation strategies, thereby increasing difficulties in managing emotional states (Path 2). When emotional distress surpasses what the individual can regulate, NSSI may emerge as a functional, though maladaptive, strategy to escape or dampen overwhelming emotional pain (Path 3). Based on this conceptual logic, we propose the following hypotheses: H1: Impulsivity serves as a mediator linking maladaptive perfectionism to NSSI; H2: Emotion regulation difficulties mediate the association between maladaptive perfectionism and NSSI; H3: Impulsivity and emotion regulation difficulties operate sequentially as a chain mediating mechanism, such that maladaptive perfectionism is associated with heightened impulsivity, which subsequently contributes to greater emotion regulation difficulties, ultimately elevating NSSI risk. , , 

This study aims to explore the psychological mechanisms underlying the relationship between impulsivity, emotion regulation difficulties, and maladaptive perfectionism in NSSI. We hypothesize that impulsivity plays a mediating role in the association between maladaptive perfectionism and NSSI. Using machine learning techniques and structural equation modeling, we investigate how these factors interact and mediate NSSI risk. This research seeks to deepen our understanding of the psychological processes linking maladaptive perfectionism and self-injurious behavior and inform the development of more effective interventions for adolescents at risk of NSSI.

## 2 Methods and materials

### 2.1 Participants

The sample for this study was drawn from five secondary schools in Gansu and Shaanxi provinces, China, representing a combination of urban and rural educational settings. A total of 4,000 questionnaires were distributed. Participants ranged in age from 13 to 19 years, with a mean age of 15.29 (SD = 1.58); 2,036 were male and 1,829 were female. To provide additional socioeconomic context, we collected information on parental education and family affluence. Approximately 58% of the sample resided in rural areas. In terms of parental education, 41% of fathers and 45% of mothers had completed secondary education or above. These demographic indicators were included as control variables in the subsequent analyses to account for their potential influence. The study was reviewed and approved by the Ethics Committee of the School of Psychology at Northwest Normal University (20240889), and informed consent was obtained from all participating students and their guardians.

### 2.2 Measures

#### 2.2.1 NSSI

NSSI was assessed using two complementary scales. The Inventory of Statements About Self-Injury (ISAS; 25) was used to measure the lifetime frequency of 12 specific NSSI behaviors (e.g., cutting, hitting). The Functional Assessment of Self-Mutilation (FASM; 26; Chinese version by 27) was used to assess 22 potential motives or functions for self-injury (e.g., to relieve negative feelings). The original scale theoretically distinguishes four functional dimensions: automatic negative reinforcement (e.g., relieving unbearable negative emotions), automatic positive reinforcement (e.g., generating certain sensations or emotions), social negative reinforcement (e.g., escaping undesirable social interactions or demands), and social positive reinforcement (e.g., seeking attention or influencing others).It is important to clarify that the ISAS and FASM serve different measurement purposes in this study. The ISAS frequency score served as the dependent variable for binary classification (presence vs. absence of NSSI) in the machine learning models and also as the observed indicator of NSSI behavior level in the structural equation modeling (SEM). In contrast, the FASM total function score was conceptualized as a psychological construct reflecting the intensity of motivational drivers behind NSSI and was included solely as a mediator in the SEM analysis. To preserve measurement specificity and avoid redundancy with the ISAS behavioral frequency assessment, only the functional subscale of the FASM was used in this study. This study used the FASM total score based on the following considerations: (1) The primary objective of this study is to initially test a relatively complex chain mediation model involving multiple variables, and using the total score helps maintain model parsimony and identifiability under theoretical guidance; (2) In preliminary analyses conducted for this study, the functional dimensions showed moderate to high intercorrelations (r = 0.68-0.82), suggesting the possible existence of a higher-order factor reflecting “overall functional severity,” for which the total score can serve as a valid indicator; (3) This approach has been widely adopted and accepted in numerous exploratory mechanism studies primarily aimed at examining overall relationships between variables. Both scales demonstrated excellent internal consistency in our sample (ISAS Cronbach’s α = 0.858; FASM Cronbach’s α = 0.945).

#### 2.2.2 Frost multidimensional perfectionism scale

The Frost Multidimensional Perfectionism Scale, originally developed by Frost (6) and later revised by Zi and Zhou (28), was used to assess the cognitive, emotional, and behavioral characteristics associated with perfectionism. The scale consists of 27 items across five dimensions: *Concern Over Mistakes* (CM), *Organization* (OR), *Parental Expectations* (PE), *Personal Standards* (PS), and *Doubts About Actions* (DA). Items are rated on a five-point Likert scale, ranging from 1 (“not at all true”) to 5 (“completely true”). In the FMPS framework, CM, DA, PS, and PE are categorized as indicators of maladaptive (negative) perfectionism, while OR reflects adaptive (positive) perfectionism. Scores for each dimension were calculated separately for analysis. In this study, the overall Cronbach’s alpha coefficient was 0.844, and the reliability coefficients for the five dimensions were 0.907, 0.880, 0.831, 0.842, and 0.794, respectively.

#### 2.2.3 Barratt impulsiveness scale

The Chinese version of the Barratt Impulsivity Scale (BIS), revised by Li et al. (29), was used in this study to assess impulsivity. The scale consists of 30 items covering three dimensions—attentional impulsiveness, motor impulsiveness, and non-planning impulsiveness. Each item is rated on a four-point scale from 1 (“never”) to 4 (“always”), with higher scores indicating greater impulsivity. The structure of the Chinese revision closely aligns with that of the original instrument, and prior research has reported internal consistency coefficients ranging from 0.56 to 0.76 across the total scale and subscales. In the present study, the overall Cronbach’s alpha coefficient was 0.884, and the reliability coefficients for the three subscales were 0.931, 0.897, and 0.886, respectively.

#### 2.2.4 Difficulties in emotion regulation scale

The Difficulties in Emotion Regulation Scale (DERS), developed by Gratz and Roemer (30), was used to assess challenges in emotion regulation. The scale contains 36 items spanning six dimensions: difficulty accepting emotional responses, difficulty engaging in goal-directed behavior, difficulty controlling impulses, limited emotional awareness, limited access to effective regulation strategies, and difficulty understanding emotions. Items are rated on a five-point Likert scale ranging from 1 (“rarely”) to 5 (“almost always”), with higher scores indicating greater difficulties in emotion regulation and lower overall regulatory capacity. In the present study, the overall Cronbach’s alpha coefficient for the scale was 0.753.

### 2.3 Statistical analysis

This study employed a complementary dual analytical strategy to jointly reveal NSSI risk factors and mechanisms. The machine learning (ML) component focuses on out-of-sample prediction accuracy and variable importance ranking, aiming to build an effective classifier for distinguishing NSSI risk and identifying the most powerful predictive features from a data-driven perspective. The structural equation modeling (SEM) component focuses on testing *a priori* theoretical hypotheses and quantifying mediation pathways between variables, aiming to validate the effectiveness of our proposed “perfectionism-impulsivity-emotion regulation-NSSI” theoretical model. The two form a logical closed loop: ML results provide empirical evidence supporting variable selection for SEM, while SEM provides theoretical explanation and mechanism interpretation for the relationships between important variables identified by ML.

#### 2.3.1 Data preprocessing and preliminary analysis

First of all, SPSS 27.0 is used to clean up the extreme values, distribution patterns, missing values, and so on. Then, SPSS 27.0 was used to perform descriptive and correlation analyses of the variables studied.

#### 2.3.2 Machine learning

We used machine learning to examine the predictive value of maladaptive perfectionism, impulsivity, and emotion regulation difficulties for NSSI. Support vector machine (SVM) analyses were performed using Python’s scikit-learn library, with all continuous variables standardized using Z-score normalization. Model training relied on ten-fold cross-validation, and hyperparameters were tuned through a grid search (C values tested: 0.1, 1, 10; kernel functions: ‘linear’ and ‘rbf’; final model: RBF kernel, C = 1.0, gamma = ‘scale’). To ensure that selecting SVM was empirically justified, we additionally compared its performance with random forests (mean AUC = 0.77) and logistic regression (mean AUC = 0.75), and SVM achieved the highest AUC (see **Table 1** for full comparisons). Feature importance was evaluated using permutation importance. To assess potential overfitting, we examined the performance gap between training and validation folds across the ten-fold cross-validation procedure, which remained below 3%. Our analysis included two tasks: classification (distinguishing individuals with vs. without NSSI) and regression (predicting the severity of NSSI behaviors).

Performance Metrics: We evaluated our models using standard metrics. For classification, we report accuracy, sensitivity, specificity, and the Area Under the Curve (AUC). The AUC measures the model’s ability to discriminate between groups, ranging from 0.5 (no better than chance) to 1.0 (perfect discrimination). For regression, we report the mean absolute error (MAE) and the coefficient of determination (R²).

#### 2.3.3 Intermediary effect analysis

Data analysis was performed using Mplus 8.3. Before conducting the path analysis, several preliminary assessments were undertaken to ensure data validity. Based on the important predictors identified through machine learning, we constructed a chain mediation model to test three specific pathways. Given the cross-sectional nature of the design, we employ cautious terminology such as “association” and “statistical mediation pattern” in result interpretation, avoiding causal inference. Path analyses examined the relationships among NSSI and its associated variables, with impulsivity as the first mediator and emotional regulation difficulties as the second. Specifically, the model tested the effect of maladaptive perfectionism on impulsivity, which, in turn, influenced emotional regulation difficulties, culminating in NSSI.

For overall model assessment, fit indices were evaluated using standard criteria: Comparative Fit Index (CFI) > 0.90, Tucker-Lewis Index (TLI) > 0.90, Root Mean Square Error of Approximation (RMSEA) < 0.08, *χ*²/df < 2.0, and Standardized Root Mean Square Residual (SRMR) < 0.05. The significance of model paths was checked by reporting beta coefficients (β). Indirect effects were tested using bootstrap methods in Mplus to estimate 95% confidence intervals. Indirect effects were considered significant if the 95% confidence interval did not include zero, based on bias-corrected bootstrap confidence intervals and standard error estimates (Bootstrap sample size = 1000) (31).

## 3 Results

### 3.1 Basic information on the variables and preliminary results of the analyses

Of the 3,865 participants, 1,287 (33.30%) reported a history of self-injury. Among these individuals, 1,115 (28.84%) had engaged in self-injurious behaviors within the past 12 months. The mean age of onset for NSSI was 13.44 years (SD = 1.70) for both males and females. The five most commonly reported forms of self-injury were cutting (709; 63.59%), smacking or hitting oneself (647; 60.45%), biting oneself (790; 70.85%), interfering with wound healing (714; 60.04%), and pinching (699; 62.69%). Descriptive statistics and correlations among the variables included in the model are presented in **Tables 1**, **2** (see **Tables 1**, **2**).

**Table 1:** Demographic information of participants (*N*  = 3865).

Variables	NSSI group	HC group	*t/x2*	*p Value*	*φ/rpb*	*p Value*
Sex (%)	43.32(M)	56.47(M)	55.069	<.001	0.119	<.001
Sample size(N)	1115	2750	NA	NA	NA	NA
Age(yrs)	15.57(1.57)	15.18(1.57)	6.903	<.001	0.110	<.001
Single Child(%)	25.2(Y)	27.78(Y)	2.677	0.102	-0.026	0.102
Residential Address(%)	40.18(C)	42.51(C)	1.770	0.183	0.021	0.183
Board(%)	55.43(Y)	56.36(Y)	0.283	0.595	-0.009	0.595
Children Left Behind(%)	9.24(Y)	6.36(Y)	9.817	0.002	0.050	0.002
Parental Marriage(%)	81.35(F)	85.42(F)	10.084	0.018	0.051	0.018
Seek treatment for mental health issues(%)	14.35(Y)	3.45(Y)	152.821	<.001	0.199	<.001
Parental health status(%)	80.72(Y)	89.27(Y)	50.694	<.001	-0.115	<.001

NSSI, Non-suicidal self-injury; HC, healthy controls; M; male; Y; yes; C; city; F, first wife.

**Table 2:** Correlation coefficients for each variable.

Item	*r*				
Variables	1	2	3	4	5
1. NSSI type	—				
2. NSSI function	.644**	—			
3. Emotion regulation difficulties	.509**	.488**	—		
4. Maladaptive perfectionism	.302**	.300**	.469**	—	
5. Impulsivity	.249**	.209**	.408**	.082**	—

**p < 0.01.

### 3.2 Common method bias test

Harman’s single-factor test for common method bias was conducted using SPSS 27.0. The results showed that 19 factors had eigenvalues greater than 1, with the first factor accounting for 20.80% of the total variance—well below the commonly accepted threshold of 40%. These findings indicate that common method bias was not a significant concern in this study (32).

### 3.3 Correlation analysis results of maladaptive perfectionism, impulsivity, emotion regulation difficulties, and NSSI

The results of the correlation analyses are presented in **Table 1**. As shown, maladaptive perfectionism was positively associated with impulsivity (r = 0.082, p = 0.001). Impulsivity was significantly correlated with emotion regulation difficulties (r = 0.408, p = 0.001). NSSI type was significantly associated with maladaptive perfectionism (r = 0.302, p = 0.001), and NSSI function was similarly correlated with maladaptive perfectionism (r = 0.300, p = 0.001). In addition, NSSI type was strongly associated with NSSI function (r = 0.644, p = 0.001). Significant correlations were also observed among different NSSI subtypes.

Maladaptive perfectionism, impulsivity, and emotion regulation difficulties showed differential correlations with NSSI type (r = 0.249–0.509, all ps = 0.001) and with NSSI function (r = 0.209–0.488, all ps = 0.001). Furthermore, age and parental health were significantly associated with maladaptive perfectionism, impulsivity, emotion regulation, and NSSI type and function (age: r = 0.050–0.199, p = 0.001–0.002; parental health: r = 0.060–0.108, p = 0.001). Sex was significantly related to maladaptive perfectionism, emotion regulation, and both NSSI type and function (r = 0.055–0.092, p = 0.001).

Given these associations, age, sex, and parental health were controlled for in subsequent analyses involving maladaptive perfectionism, impulsivity, emotion regulation, and NSSI indicators. Hierarchical regression analyses were conducted to examine the predictive effects of maladaptive perfectionism and emotion regulation on NSSI type and function. The analysis proceeded in four steps: sex, age, and parental health were entered in the first block; maladaptive perfectionism or impulsivity in the second; emotion regulation in the third; and NSSI in the fourth. As shown in **Table 2**, maladaptive perfectionism accounted for 11.5% and 10.4% of the variance in NSSI type and function, respectively. Impulsivity accounted for 15.6% and 13.3%, while emotion regulation difficulties explained 27.2% of the variance in NSSI type and 24.8% in NSSI function (see **Table 3**).

**Table 3:** Hierarchical regression analysis for predicting the type and function of NSSI.

Predictor variable	NSSI type			NSSI function			
	*β*	*B(SE)*	*ΔR²*	*β*	*B(SE)*		*ΔR²*
step 1			0.033***			0.022***	
genders	0.135***	0.727(0.085)		0.086***	1.224(0.226)		
age	0.054***	0.923(0.27)		0.097***	0.434(0.072)		
Parental health status	-0.097***	-2.434(0.399)		-0.066***	-1.379(0.335)		
step 2			0.082***			0.082***	
maladaptive perfectionism	0.288***	0.13(0.007)		0.288***	0.108(0.006)		
step 3			0.041***			0.029***	
impulsivity	0.207***	0.114(0.008)		0.175***	0.081(0.007)		
step 4			0.116***			0.115***	
emotion regulation	0.43***	0.171(0.007)		0.428***	0.142(0.006)		

NSSI, Non-suicidal self-injury.

***p < 0.001.

### 3.4 Machine learning results

**Figure 2:**
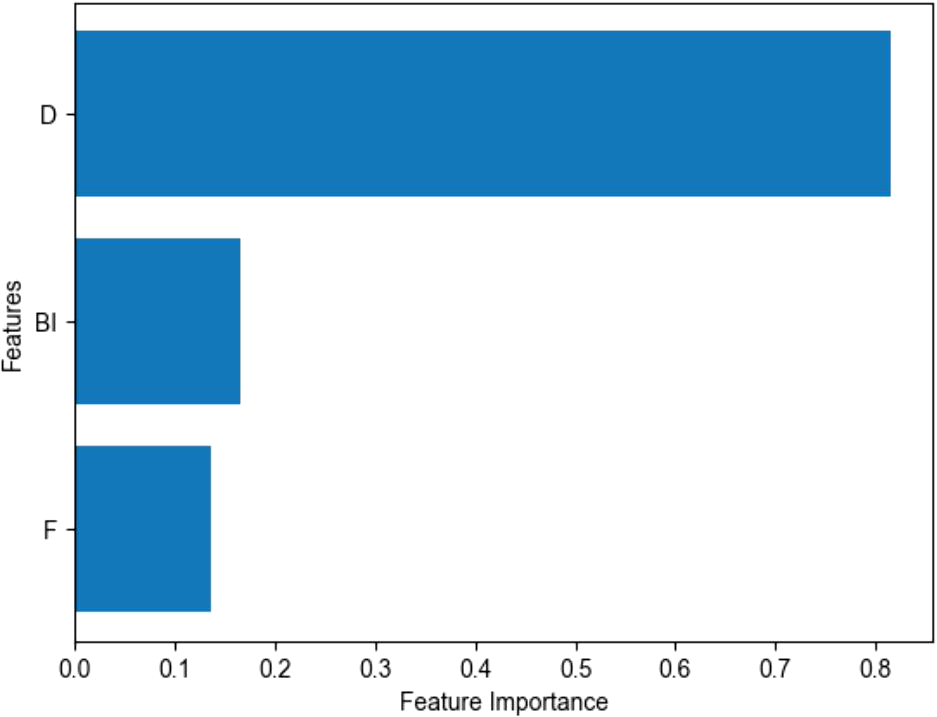
Feature Importance using Linear SVC. F: CFMPS, measuring the cognitive, emotional, and behavioral characteristics typical of perfectionists, BI, BIS-11, measuring Impulsiveness, D, DERS, measuring Difficulties in Emotion Regulation.

**Figure 3:**
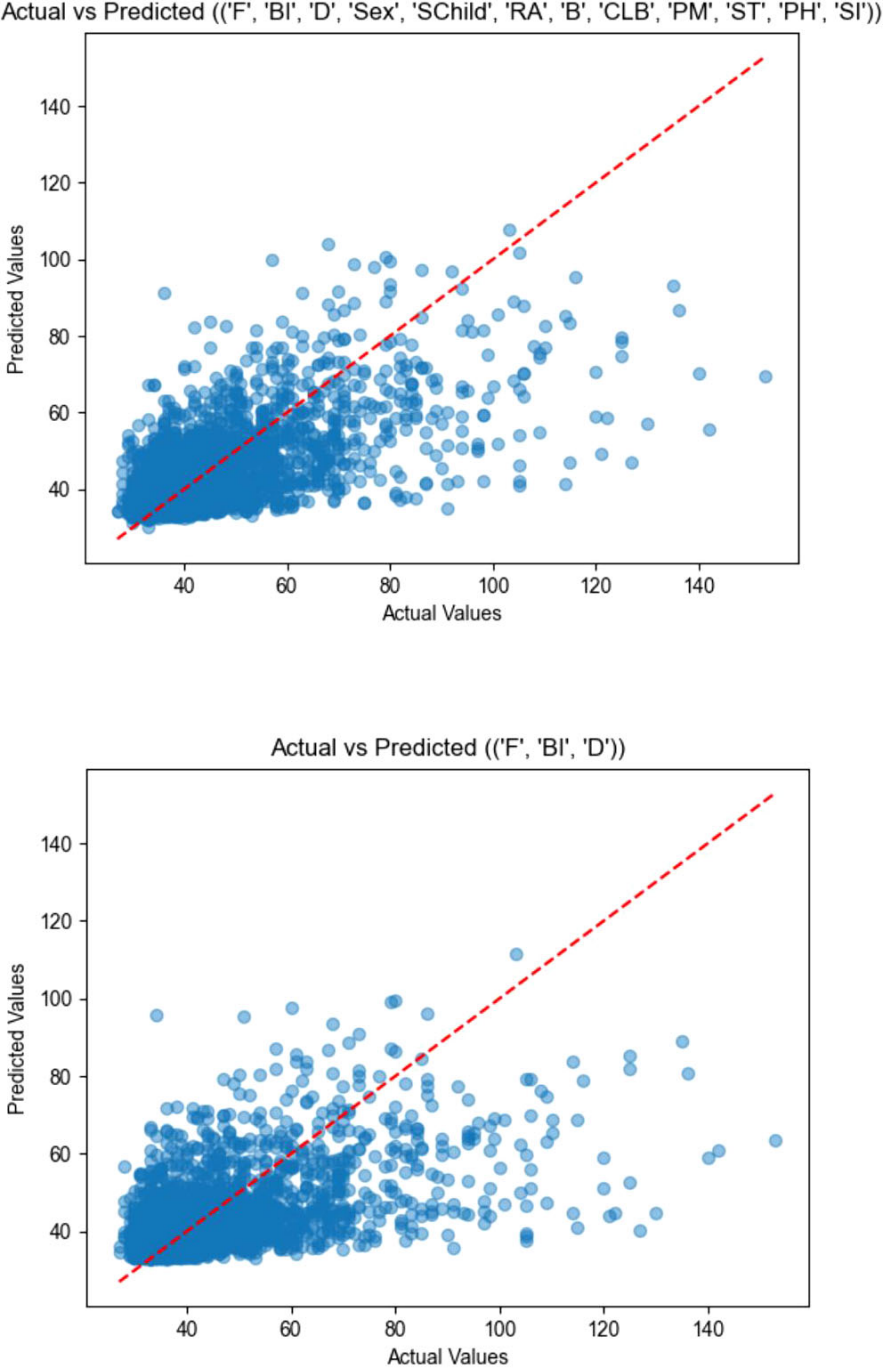
A scatter plot between actual and predicted values. Actual vs Predicted [('F', 'BI', 'D', 'Sex', 'SChild', 'RA', 'B', 'CLB', 'PM', 'ST', 'PH', 'SI')] F: CFMPS, measuring the cognitive, emotional, and behavioral characteristics typical of perfectionists, BI, BIS-11, measuring Impulsiveness, D, DERS, measuring Difficulties in Emotion Regulation, SChild: Single Child, RA, Residential Address, B, Board, CLB, Children Left Behind, PM, Parental Marriage, PH, Parental Health, SI, suicidal ideation.

The combination of maladaptive perfectionism, impulsivity, and emotion regulation difficulties achieved the best performance in classifying adolescents with and without NSSI. The final model showed good sensitivity (0.86) and an Area Under the Curve (AUC) of 0.79. According to standard guidelines (33), an AUC between 0.7 and 0.8 is considered ‘acceptable’ to ‘good’ discrimination (see in **Figure 1**). In practical terms, this indicates that a screening tool based on these three psychological factors would be substantially more effective than random chance in identifying at-risk adolescents in school or community settings. It is important to note that although NSSI is the primary focus of this study, suicidal ideation (SI) was included in the predictive feature set. Conceptually, NSSI and SI are distinct: NSSI functions primarily as a means of regulating overwhelming emotional states and does not involve an intention to die, whereas SI explicitly entails thoughts of ending one’s life. Nevertheless, in clinical settings, these phenomena frequently co-occur as part of a broader self-harm spectrum. Including SI in the model allows us to statistically account for its potential confounding influence on NSSI risk. From a practical risk-assessment standpoint, it also enhances the model’s capacity to identify adolescents who may exhibit elevated overall self-harm risk and therefore require more immediate clinical attention (see in **Figures 2**, **3** and **Table 4**).

**Table 4:** The average of 10-fold cross-validation results is based on different feature combinations.

Number of features	Features	AUC	ACC	SEN	SPE	Precision	Recall	F1-score
3	('F', 'BI', 'D')	0.79	0.76	0.86	0.52	0.82	0.86	0.84
12	('F,' 'BI,' 'D,' 'Sex,' 'SChild,' 'RA,' 'B,' 'CLB,' 'PM,' 'ST,' 'PH,' 'SI')	0.74	0.76	0.86	0.52	0.81	0.86	0.84
4	('F', 'BI', 'D', 'SI')	0.78	0.76	0.85	0.54	0.82	0.85	0.83
4	('F', 'BI', 'D', 'PH')	0.73	0.74	0.88	0.39	0.78	0.88	0.83
4	('F', 'BI', 'D', 'ST')	0.74	0.74	0.86	0.45	0.79	0.86	0.83
4	('F', 'BI', 'D', 'PM')	0.78	0.76	0.86	0.49	0.81	0.86	0.83
4	('F', 'BI', 'D', 'CLB')	0.76	0.75	0.87	0.46	0.80	0.87	0.83
4	('F', 'BI', 'D', 'B')	0.79	0.76	0.86	0.52	0.81	0.86	0.84
4	('F', 'BI', 'D', 'RA')	0.79	0.76	0.86	0.52	0.86	0.86	0.84
4	('F', 'BI', 'D', 'SChild')	0.78	0.76	0.86	0.49	0.81	0.86	0.83
4	('F', 'BI', 'D', 'Sex')	0.79	0.76	0.85	0.53	0.82	0.85	0.84
2	('BI', 'D')	0.78	0.76	0.88	0.47	0.80	0.88	0.84
2	('F', 'D')	0.79	0.76	0.87	0.46	0.80	0.87	0.84
2	('F', 'BI')	0.71	0.71	0.99	0	0.71	0.99	0.83
2	('SC', 'S2')	0.75	0.74	0.92	0.31	0.77	0.92	0.84

F: CFMPS, measuring the cognitive, emotional, and behavioral characteristics typical of perfectionists; BI: BIS-11, measuring Impulsiveness; D: DERS, measuring Difficulties in Emotion Regulation; Schild: Single Child; RA: Residential Address; B: Board; CLB: Children Left Behind; PM: Parental Marriage; PH: Parental Health; SI: suicidal ideation.

### 3.5 Analysis of mediating mechanisms between maladaptive perfectionism and NSSI

**Figure 4:**
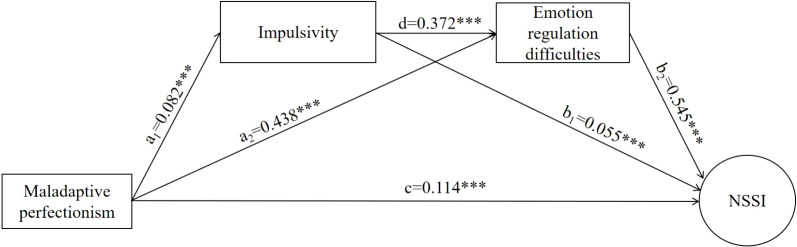
Chain-mediated path diagram of adolescent maladaptive perfectionism to NSSI. ***p < 0.001.

In this study, the chain mediation model examining the effect of maladaptive perfectionism on adolescent non-suicidal self-injury (NSSI) was tested using structural equation modeling (SEM) in Mplus 8.3. The overall model demonstrated excellent fit: *χ*²/df = 3.325, CFI = 0.999, TLI = 0.996, RMSEA = 0.025, and SRMR = 0.005. As shown in **Figure 1**, maladaptive perfectionism was significantly associated with impulsivity (*a_1_* = 0.082, *p* = 0.001). Impulsivity, in turn, was significantly associated with emotion regulation difficulties (*d* = 0.372, *p* = 0.01), and emotion regulation difficulties were positively related to NSSI (*b₂* = 0.545, *p* = 0.001). Bootstrap analyses further supported the significance of the chain mediation effect. The indirect pathway from maladaptive perfectionism → impulsivity → emotion regulation difficulties → NSSI was significant (B = 0.006, 95% CI [0.004, 0.010]), indicating that this sequential mediation pathway meaningfully contributes to the relationship (34). In addition, maladaptive perfectionism showed a significant direct association with NSSI (*c* = 0.114, *p* = 0.001). A simple mediation pathway through impulsivity also emerged: maladaptive perfectionism positively predicted impulsivity (*a_1_* = 0.082, *p* = 0.001), which subsequently showed a positive association with NSSI (*b_1_* = 0.055, *p* = 0.001). The bootstrap estimate for this indirect pathway was significant (B = 0.002, 95% CI [0.001, 0.004]), confirming the mediating role of impulsivity. Moreover, maladaptive perfectionism was significantly associated with emotion regulation difficulties (*a₂* = 0.438, *p* = 0.001), which in turn predicted greater NSSI (*b₂* = 0.545, *p* = 0.001). The bootstrap estimate for this indirect pathway was also significant (B = 0.088, 95% CI [0.080, 0.102]), indicating that emotion regulation difficulties serve as an independent mediator of the association between maladaptive perfectionism and NSSI (see in **Figure 4** and **Tables 4**, **5**).

**Table 5:** Machine learning and path analysis results summary.

Metric category	Metric	Value (95% CI)	Interpretation
Machine Learning Performance	AUC	0.79 [0.76, 0.82]	Good discrimination
	Accuracy	0.76 [0.73, 0.79]	
	Sensitivity	0.86 [0.83, 0.89]	High true positive
	Specificity	0.52 [0.48, 0.56]	Moderate true negative
	F1-score	0.78 [0.75, 0.81]	Balanced metric
	PPV	0.71 [0.68, 0.74]	Positive predictive value
	NPV	0.73 [0.70, 0.76]	Negative predictive value
Path Coefficients (Standardized)	Perfectionism → Impulsivity	β = 0.082*** [0.061, 0.103]	Small effect
	Impulsivity → Emotion Regulation	β = 0.372*** [0.349, 0.395]	Medium effect
	Emotion Regulation → NSSI	β = 0.545*** [0.521, 0.569]	Large effect

***p <.001. PPV, Positive Predictive Value; NPV, Negative Predictive Value.

## 4 Discussion

This study offers an important advancement in understanding the psychological mechanisms underlying NSSI through the innovative integration of machine learning–based prediction and structural equation modeling. As the first investigation to systematically examine the distinguishing and predictive roles of maladaptive perfectionism, impulsivity, and emotion regulation difficulties in adolescent NSSI within a Chinese cultural context, our findings extend existing theoretical frameworks and provide clinically meaningful insights to inform targeted prevention and intervention efforts.

### 4.1 The mechanism of impulsivity and emotional regulation difficulties

Our findings delineate a coherent psychological pathway from maladaptive perfectionism to NSSI. The identified chain mediation model demonstrates that maladaptive perfectionism confers vulnerability to NSSI not only directly but also through two distinct yet interconnected mechanisms: by fostering impulsive reactivity and, more profoundly, by severely compromising emotion regulation capacity. The pathway through impulsivity aligns with historical conceptualizations of NSSI as related to impulse-control deficits (35, 36). We found that the rigid, high-stakes self-evaluation inherent in maladaptive perfectionism creates a state of psychological tension that manifests as impulsive reactivity (37), characterized by a focus on short-term emotional relief that facilitates self-injurious acts.

Our results support the pivotal role of emotion regulation difficulties. Individuals with maladaptive perfectionism often struggle profoundly with emotional dysregulation (38, 39). According to the emotional dysregulation model of perfectionism (40), these individuals establish excessively high standards to preempt criticism (41). When inevitably failing to meet these standards, they engage in harsh self-criticism, triggering intense negative emotions like shame and anxiety that overwhelm their regulatory capacity, making NSSI a likely maladaptive coping strategy (42). Crucially, our chain mediation model reveals that impulsivity and emotion regulation difficulties do not operate in isolation. The path from impulsivity to emotion regulation difficulties indicates a dynamic interplay: the tendency to act rashly—particularly under conditions of negative urgency (43)—may itself disrupt the application of adaptive regulatory strategies, thereby compounding dysregulation. This creates a vicious cycle in which perfectionism-driven distress fosters impulsivity, which, in turn, undermines regulatory capacity, ultimately culminating in NSSI.

These mechanistic insights integrate powerfully with the Emotional Cascade Model (ECM) (24). We propose that maladaptive perfectionism acts as a primary catalyst for the ruminative cycles central to the ECM. The relentless self-criticism and chronic fear of failure provide a constant stream of ruminative content (40), thereby initiating and intensifying the emotional avalanches that precipitate NSSI. This is corroborated at the mechanistic level by our mediation model and powerfully underscored by the machine-learning result, which identifies emotion regulation difficulties as the top-ranking predictive feature. This convergence provides robust empirical validation of the central role of emotional dysregulation—a cornerstone not only of the ECM but also of the Experiential Avoidance Model (17), which conceptualizes NSSI as a maladaptive strategy to escape distressing inner experiences. Furthermore, this aligns with Tonta’s (44) observation that perfectionism contributes to NSSI by fueling negative emotions and rumination.

### 4.2 Cultural context and culturally-informed implementation

The findings of this study should be interpreted within the broader socio-cultural context of contemporary China, which shapes both the development of key risk factors and the ways in which NSSI is expressed. Within Chinese collectivist culture, the strong emphasis on academic achievement—coupled with deeply rooted concerns about “face”—creates substantial external pressure that may heighten adolescents’ susceptibility to maladaptive perfectionism (45). The intense academic competition and elevated parental expectations characteristic of the Chinese educational environment likely amplify the perfectionistic demands reflected in our model. In parallel, cultural norms surrounding emotional expression—particularly the tendency to suppress direct displays of negative emotion—may lead some adolescents to use NSSI as a covert means of communicating distress or regulating overwhelming affect when open emotional expression feels constrained. These cultural and psychological dynamics highlight the importance of developing culturally sensitive interventions that both acknowledge the realities of academic pressure and equip adolescents with adaptive strategies to manage perfectionistic tendencies and build emotional awareness in culturally acceptable ways.

### 4.3 Clinical implications

This study’s integration of mediation modelling and machine learning offers a precise blueprint for NSSI intervention. Our findings indicate that effective programs should simultaneously target maladaptive perfectionism, impulsivity, and emotion regulation through an integrated approach. To address maladaptive perfectionism, Cognitive-Behavioral Therapy (CBT) provides evidence-based techniques, including cognitive restructuring of perfectionistic beliefs and behavioral experiments to challenge the fear of failure (46). To counter impulsivity—particularly negative urgency—mindfulness training creates crucial cognitive space between the urge and the action, enhancing behavioral inhibition through non-judgmental awareness practice. Most critically, given that emotion regulation difficulties emerged as the primary predictor, Dialectical Behavior Therapy (DBT) skills offer essential tools (47). These include Distress Tolerance techniques (e.g., TIPP skills for crisis management) and Emotion Regulation modules for navigating intense affective states triggered by perfectionistic setbacks. In implementation, school-based prevention can integrate psychoeducation with mindfulness and core DBT skills, while clinical settings may combine comprehensive DBT with targeted CBT for perfectionism. This multi-component approach directly addresses the interconnected pathways identified in our study, providing a structured framework for reducing NSSI risk in vulnerable adolescents.

### 4.4 Theoretical integration and mechanistic elucidation

This study extends theoretical frameworks in three key dimensions, empirically refining prevailing models of self-injurious behavior. First, we identify a chain mediation pathway clarifying how maladaptive perfectionism acts as a trait-level trigger within the Emotional Cascade Model (24). The persistent self-evaluative pressure in perfectionism provides a cognitive–emotional substrate for rumination (40), fueling the emotional surges central to the ECM. Second, multi-method convergence—with emotion regulation difficulties as the most discriminative feature in machine learning and the final pathway in SEM—strengthens the Experiential Avoidance Model (17), supporting NSSI as a maladaptive response to unregulated distress. Third, results reveal a nuanced impulsivity–emotion regulation dynamic: perfectionism generates tension expressed as impulsive reactivity (37), consistent with impulse-control deficit theories (36), while rash responses under negative urgency (43) further undermine regulation. This forms a self-reinforcing loop where perfectionism heightens impulsivity, which erodes regulatory capacity, increasing NSSI vulnerability.

### 4.5 Limitations and methodologically rigorous future directions

Several limitations warrant consideration in interpreting these findings. First, the cross-sectional design precludes definitive causal inference; longitudinal studies are needed to establish temporal precedence in the proposed mediation pathways. Second, although Harman’s single-factor test results suggest that common method bias may not pose a serious threat in this study, we acknowledge that complete reliance on self-report data remains an important limitation of this research. Participants may underestimate their NSSI behavior frequency or impulsivity due to social desirability, or may be unable to accurately recall due to memory bias. Meanwhile, shared method variance may potentially inflate correlations between variables. Future research urgently needs to adopt multi-informant reports (such as parent, teacher, or clinician assessments) combined with more objective behavioral tasks (e.g., Go/No-Go tasks measuring impulsivity) or physiological indicators (e.g., heart rate variability monitoring emotional responses) to achieve more robust and comprehensive measurement of core constructs. Third, the exclusively Chinese sample limits generalizability; cross-cultural replications are essential to test the universality and boundary conditions of our model across diverse cultural contexts. Finally, while key demographics were controlled, more nuanced socioeconomic measures and additional risk-protective factors should be examined to build a more comprehensive understanding of NSSI vulnerability.

In conclusion, this study demonstrates that maladaptive perfectionism, impulsivity, and emotion regulation difficulties operate within an interconnected psychological system that increases vulnerability to NSSI. The innovative combination of machine learning and structural equation modeling offers both predictive validation and mechanistic insight into this pathway, pointing toward new avenues for developing targeted, multi-component interventions for adolescents at heightened risk of self-injury.

## Data Availability

The raw data supporting the conclusions of this article will be made available by the authors, without undue reservation.
